# Quantitative Proteomic Analysis Reveals That Arctigenin Alleviates Concanavalin A-Induced Hepatitis Through Suppressing Immune System and Regulating Autophagy

**DOI:** 10.3389/fimmu.2018.01881

**Published:** 2018-08-16

**Authors:** Qin Feng, Jingchun Yao, Ge Zhou, Wenkai Xia, Jingang Lyu, Xin Li, Tao Zhao, Guimin Zhang, Ningwei Zhao, Jie Yang

**Affiliations:** ^1^State Key Laboratory of Pharmaceutical Biotechnology, School of Life Sciences, Nanjing University, Nanjing, China; ^2^Center for New Drug Pharmacological Research of Lunan Pharmaceutical Group, State Key Laboratory, Generic Manufacture Technology of Chinese Traditional Medicine, Linyi, China; ^3^Affiliated Hospital of Nanjing University of Chinese Medicine, Nanjing, China; ^4^School of Pharmacy, Linyi University, Linyi, China; ^5^Shimadzu Biomedical Research Laboratory, Shanghai, China; ^6^State Key Laboratory of Proteomics, Beijing Proteome Research Center, Beijing Institute of Radiation Medicine, Beijing, China

**Keywords:** arctigenin, concanavalin A, IFN-γ, Stat1, immune system, autophagy, apoptosis, proteomics

## Abstract

Concanavalin A-induced autoimmune hepatitis is a well-established experimental model for immune-mediated liver injury. It has been widely used in the therapeutic studies of immune hepatitis. The in-depth analysis of dysregulated proteins from comparative proteomic results indicated that the activation of immune system resulted in the deregulation of autophagy. Follow-up studies validated that some immune related proteins, including Stat1, Pkr, Atg7, and Adrm1, were indeed upregulated. The accumulations of LC3B-II and p62 were confirmed by immunohistochemistry and Western blot analyses. Arctigenin pretreatment significantly alleviated the liver injury, as evidenced by biochemical and histopathological investigations, whose protective effects were comparable with Prednisone acetate and Cyclosporin A. Arctigenin pretreatment decreased the levels of IL-6 and IFN-γ, but increased the ones of IL-10. Next, the quantitative proteomic analysis demonstrated that ARC pretreatment suppressed the activation of immune system through the inhibition of IFN-γ signaling, when it downregulated the protein expressions of Stat1, P-Stat1, Pkr, P-Pkr, Bnip3, Beclin1, Atg7, LC3B, Adrm1, and p62. Meanwhile, Arctigenin pretreatment also reduced the gene expressions of Stat1, Pkr, and Atg7. These results suggested that Arctigenin alleviated autophagy as well as apoptosis through inhibiting IFN-γ/IL-6/Stat1 pathway and IL-6/Bnip3 pathway. In summary, the comparative proteomic analysis revealed that the activation of immune system led to Concanavalin A-induced hepatitis. Both autophagy and apoptosis had important clinical implications for the treatment of immune hepatitis. Arctigenin might exert great therapeutic potential in immune-mediated liver injury.

## Introduction

Concanavalin A (ConA) is a plant lectin from seeds of Jack beans (*Canavalia ensiformis*). Intravenous injection of ConA leads to CD4^+^ T cell-mediated hepatitis in mice (1). The model might allow the study of the pathophysiology of self- or foreign antigen-mediated hepatic failure such as autoimmune hepatitis (AIH) and viral hepatitis (2). Some cytokines, including interferon (IFN)-γ and tumor necrosis factor (TNF)-α (3, 4), might participate in this process (3, 4). It has been shown that the alleviation of Con A-induced hepatitis was observed in IFN-γ^−/−^ mice but not in TNF-α^−/−^ mice, suggesting that IFN-γ rather than TNF-α is a key regulator in Con A-induced liver injury (5). During viral infections, the antiviral effect of IFN-γ was stronger and more durable than the ones of IFN-α and IFN-β (6). The class of genes and proteins, which were predominantly upregulated by IFN-γ, were directly involved in the activation of the immune system, including antigen processing and presentation, recruitment of T cells and attack against the virus-infected hepatocytes (7). The killing of virus is accompanied with the apoptosis of liver cells, and apoptosis is the main mechanism of hepatitis (8, 9).

IFN-γ mediates apoptosis *via* activation of Stat1 (10, 11). IFN-γ activates Stat1 to form homodimers that bind to IFN-γ activated sequence (GAS) on the promoter, so as to activate IFN-γ-induced gene expression (12). Transgenic mice overexpressing Stat1 showed elevated levels of IFN-γ and significantly aggravated liver injury after ConA administration, while Con A-induced liver injury hardly occurred in Stat1^−/−^ mice (13). This is due to Stat1-mediated upregulation of IFN-γ, which enhances the production of chemokines, adhesion molecules, and ROS (14, 15). As reported, autophagy can facilitate IFN-γ-induced cellular inflammation *via* the activation of Jak2–Stat1 signaling (16). Atg5 deficiency extremely inhibited the IFN-γ-induced pro-inflammatory responses (17).

Autophagy is a highly conserved lysosomal degradation pathway that regulates cellular homeostasis and disposes intracellular pathogens in eukaryotic cells (18, 19). The stages of autophagy include induction, phagophore formation, autophagosome formation and maturation, autolysosome formation, and final degradation (20). Autophagy is initiated by the activation of the unc-51-like kinase 1 (ULK1; Atg1 in yeast) complex. Then, PI3K (Vps34 in yeast), beclin 1 (Atg6 in yeast), VPS15 (PIK3R4), and Atg14L (Atg14) form the class III phosphatidylinositol 3-kinase complex to trigger vesicle nucleation. The Atg12-Atg5-Atg16 complex promotes phagophore elongation by conjugation of Atg8 to phosphatidylethanolamine (PE). The process is mediated by Atg7 and Atg3. LC3 (Atg8 in yeast) is widely used a marker for autophagosomes. During autophagy, LC3 is processed by the removal of the C-terminal 22 amino acids to form LC3-I, followed by conjugation with PE to become LC3-II. The amount of LC3-II is widely used for the quantification of autophagosome formation (21). Once the autophagosome was formed or enhanced, a blockage of autophagic flux at late steps will downregulate the clearance of autophagosomes, as reflected by the accumulation of the autophagic substrate SQSTM1/p62. A blockage of autophagic flux finally results in autophagy-dependent cell death (22).

Evidences showed that accumulation of autophagosomes were easily observed under the electron microscope in ConA-induced hepatitis mice model, and the upregulation of Beclin1 and LC3 confirmed the observation stated above (23–25). Autophagic cell death can be observed in hepatocytes as well as endothelial cells (26). The deregulated autophagy was also found in biliary epithelial lesion in primary biliary cirrhosis (27). The inhibition of autophagy may represent a new therapy for AIH. Many natural products were reported to attenuate liver injury by suppressing autophagy as well as apoptosis in ConA-induced hepatitis, such as quercetin (24), astaxanthin (25), shikonin (28), epigallocatechin-3-gallate (29), and resveratrol (30).

Arctigenin (ARC), a phenylpropanoid dibenzylbutyrolactone lignin, is a biologically active lignan extracted from the seeds of *Arctium lappa* L. (Compositae). ARC was shown to have distinct antioxidant and anti-inflammatory properties. It exhibited protective properties on several inflammatory diseases, including brain damage (31), neurotoxicity (32–34), cardiovascular diseases (35), kidney injury (36), colitis (37), encephalomyelitis (38), asthma (39), and lung injury (40). ARC can inhibit the T lymphocyte proliferation, Th17 differentiation, macrophage activation and the release of pro-inflammation cytokines (37, 41, 42). The anti-inflammation mechanisms of ARC included the suppressions of MAPK, NF-κB, Stat1 and iNOS *via* the activation of AMPK (40, 42, 43). All these data suggest that ARC is likely to be used as a regulator of immune-mediated diseases. In addition, ARC has potent antivirus activity *in vitro* and *in vivo* (44–46). However, to our knowledge, the potential protective effects of ARC have not been evaluated in immune-mediated hepatitis *in vivo*. The autophagy involved in pathogenesis of ConA-induced hepatitis, there is still need evidence to confirm this. The proteomic methods were extremely valuable to study organ responses and to elucidate mechanism of disease and drugs by monitoring the changes of protein expressions (47). The quantitative proteomic analysis using ^12^C6- or ^13^C6-NBS (2-nitrobenzenesulfenyl) labeling followed by MALDI-TOF mass spectrometric analysis has shown its superiority for the scope and accuracy of mass spectrometry-based proteomics studies (48). Thus, quantitative proteomic analysis based on NBS labeling of peptides and nano-LC/MALDI-TOF MS were used to elucidate the mechanism of ConA-induced hepatitis. On the basis of proteomic data, the mechanisms hidden behind the protective effects of ARC on hepatitis were further investigated.

## Materials and Methods

### Materials

Arctigenin was provided by State Key Laboratory, Generic Manufacture Technology of Chinese Traditional Medicine, Lunan Pharmaceutical Group, purity >99%, white powder. MCT, HS15, labrasol, and transcutol were provided by BASF. ConA was purchased from Sigma-Aldrich (St. Louis, MO, USA). Anti-Apg7 antibody (ab133528), Anti-SQSTM1/p62 antibody (ab91526), and Anti-ADRM1 antibody [EPR11450(B)] (ab157218) was purchased from Abcam, USA. The antibodies against LC3B, Beclin1, Stat1, p-Stat1 (Y701), Pkr, p-Pkr (T446), and Bnip3 were from Immunoway, USA. Cyclosporin A oral solution (CSA) was provided by Lunan Houpu Pharmaceutical Co., Ltd. Prednisone acetate (PS) was purchased from Zhejiang Xianju Pharmaceutical Co., Ltd.

### Animals and Experimental Design

Female Balb/c mice weighing between 18 and 22 g (5–6 weeks old) were purchased from Vital River Laboratory Animal Co., Ltd. (Beijing, China). The mice were housed in a clean room at a temperature of 23 ± 2°C and a humidity of 50 ± 5% with a 12 h alternating light and dark cycle. They were permitted free access to food and water. All animal experiments were performed according to the National Institutes of Health Guidelines for the Care and Use of Laboratory Animals and were approved by the Animal Care and Use Committee of Nanjing University, China.

ConA was dissolved in pyrogen-free normal saline (NS) and injected intravenously at the dose of 10, 15, and 20 mg⋅kg^−1^ to monitor survival rate. ConA was injected intravenously at the dose of 15 mg⋅kg^−1^ to monitor the effects of different doses of ARC on survival rate. ARC was dissolved in emulsion (MCT:HS15:labrasol:transcutol 1:3:1:1) (12.5, 25, and 50 mg⋅ml^−1^) and diluted with distilled water to 0.25, 0.5, and 1 mg⋅ml^−1^ for use. 2% emulsion was used as vehicle. The ARC was administrated by intragastric at a dose of 2.5, 5, and 10 mg⋅kg^−1^. The model group was administered with vehicle, the drugs were given twice per day for 10 days. The survival rates of mice were monitored continuously for 7 days, but no more mice died after 24 h.

ConA was injected intravenously at the dose of 10 mg⋅kg^−1^ to study the protective mechanism of drug. The ARC and CSA were administered by intragastric at a dose of 10 mg⋅kg^−1^. PS was dissolved in distilled water and was administrated by intragastric at a dose of 5 mg⋅kg^−1^. Finally, the mice were killed at the indicated time points after ConA injection, and their serum and liver tissue samples were collected. The right lobe of liver was fixed in 10% formalin for morphological analysis. Remaining liver tissues were stored at −80°C for further analysis.

### Serum Biochemical Analysis

The plasma alanine transaminase (ALT), aspartate transaminase (AST) activities, total bilirubin (TBIL), and lactate dehydrogenase (LDH) were detected by BS-800 automatic biochemistry analyzer (Shenzhen Mindray Bio-Medical Electronics CO., Ltd., China). The animals were killed after anesthetization with an intraperitoneal injection of sodium pentobarbital (50 mg⋅kg^−1^).

### Measurements of Cytokines

The CBA Flex Set (BD Biosciences, USA) was used for simultaneous detection of serum IL-6, IFN-γ, and IL-10 concentration in serum according to the manufacturer’s instructions. The samples were measured with the CytoFLEX flow cytometer (Beckman Coulter Life Sciences), and the data were analyzed by the CytExpert software (Beckman Coulter Life Sciences).

### Protein Sample Preparation

The liver samples were subjected to proteomic analysis. Liver samples were prepared using Ready Prep Protein Extraction kit (Bio-Rad) at first. Extracted protein concentration was determined by BioSpec-nano (Shimadzu Biotech, Kyoto). Approximately 4 mg of protein/group was used for quantitative proteomic profiling. NBS tagging was performed according to the manufacturer’s protocol (13CNBS stable isotope labeling kit; Shimadzu). Briefly, each solution (each containing 400 µg of protein) was labeled with isotopically ^12^C6NBS or ^13^C6NBS reagent. NBS-tagged proteins were then mixed, reduced, alkylated, and digested by trypsin. NBS-tagged peptides were enriched and separated by 2D-nano HPLC (Prominence HPLC, Shimadzu) as described previously (49). Eluates were automatically deposited onto MALDI target plates by the LC spotting system (AccuSpot; Shimadzu Biotech, Kyoto). These spotted samples were automatically analyzed by MALDI-TOF/TOF MS (MALDI-7090, Shimadzu Kratos, Manchester, UK).

### Relative Quantification and Identification of Liver Tissue Proteome

Relative quantification between ^12^C6NBS- or ^13^C6NBS-tagged peptides was performed using the proteome analysis assistant software for relative quantification, TWIP Version 1.0 (DYNACOM, Chiba, Japan), referring to a monoisotopic mass list from MASCOT Distiller Ver. 1.1.2 (Matrix Science, London, UK). Threshold values of ^13^C6/^12^C6 ratios in NBS-tagged peptide pairs were set to either larger than 1.25 or less than 0.8. Candidate peptides having significant difference in peptide pair ratios were selected and further subjected to MS/MS analysis. Proteins were identified by MASCOT MS/MS Ion Search algorithm (Version 2.0; Matrix Science) using mass lists generated by MASCOT Distiller. The Mascot search parameters were as follows: trypsin digestion allowing up to 1 missed cleavages, fixed modifications of ^12^C6NBS (or ^13^C6NBS) (W) and carbamidomethyl (C), variable modifications of oxidation (M), peptide tolerance of 0.2 Da, and MS/MS tolerance of 0.8 Da. Search results with *p* values less than 0.05 were judged as positive identifications.

### Histopathology and Immunohistochemistry

Liver injury was assessed by light microscopy. Fixed liver tissue slices were processed and embedded in tissue embedding rings (Tissue-Tek^®^ TEC™ 5 Sakura Fine tek Japan CO., Ltd.). Sections of 4 µm in thickness were subjected to hematoxylin and eosin staining by automatic staining machine (Leica ST5020). Paraffin sections (4 μm) were carried out for immunohistochemistry in the liver. Briefly, sections were deparaffinized and rehydrated using automatic staining machine (Leica TP5020). Citric acid buffer (PH 6.0) in high pressure was used for antigen retrieval. Peroxidase blocking was performed using 3% hydrogen peroxide for 10 min. Sections were incubated with different antibodies respectively for 12 h at 4°C. Thereafter, sections were incubated using anti-murine/rabbit Universal Immunoperoxidase Polymer kit (Proteintech Group). Sections were counterstained with hematoxylin. Slides were viewed and captured by Pannoramic Viewer1.15.4 and analyzed with Image pro plus (IPP) 6.0. The IHC staining within each lesion was assessed by estimating the area of the objects plus the average intensity of per object, as the integrated optical density (IOD).

### Western Blot Analysis

Extracted samples containing 100 µg protein were separated by 10% (w/v) sodium dodecyl sulfate-polyacrylamide gel electrophoresis, and electrophoretically transferred onto a polyvinylidene difluoride transfer membrane. The nonspecific sites were blocked with a solution containing 5% non-fat milk powder in TBS with 0.1% Tween 20 (TBST) for 2 h at room temperature and then incubated with antibodies in TBST containing 5% bovine serum albumin overnight at 4°C with gentle shaking. After the membrane was washed, it was incubated with horseradish peroxidase-conjugated second antibodies (anti-rabbit IgG at a dilution of 1/5,000) at room temperature for 1 h. Protein bands were visualized using chemiluminescence reagent. β-actin was utilized as a housekeeping protein here.

### RT-PCR Analysis

The RT-PCR analysis was performed as described previously (50). Total RNA was extracted from liver tissue with Trizol reagent as described by the manufacturer (Gibco). RT-PCR was performed using the Access RT-PCR Introductory System (Promega) with indicated primers (Table 1). PCR was performed for 30 cycles in 25 µL of reaction mixture. PCR products were monitored using microchip electrophoresis system (MultiNA, Shimadzu Biotech, Kyoto). β-actin was utilized as a housekeeping gene here.

**Table 1:** Nucleotide sequences of primers used for RT-PCR.

Gene		Primer sequence (5′→3′)
IL-6	Forward Reverse	GGCCTTCCCTACTTCACAAG ATTTCCACGATTTCCCAGAG
IFN-γ	Forward Reverse	CAAGTGGCATAGATGTG GAA CTGGACCTGTGGGTTGTT
IL-10	Forward Reverse	ATTTGAAT TCCCTGGGTGAGAAG CACAGGGGAGAAATCGATGACA
Pkr	Forward Reverse	GTACAAGCGCTGGCAGAACTCAAT AAGAGGCACCG GGTTTTGTAT
Stat1	Forward Reverse	TGGGGGAGGGGCCTTCTTGATG TGGCCCCCTTAATGGATGTGCAA
Atg7	Forward Reverse	ATAT CGCTGCGCTGGTCGTC TGATGGAGCAGGGTAAGACC
β-actin	Forward Reverse	ATAT CGCTGCGCTGGTCGTC TGATGGAGCAGGGTAAGACC

### Data and Statistical Analysis

All experimental data obtained from mice were expressed as mean ± SD. Statistical significance was determined by Student’s unpaired two-tailed *t*-test. *P* < 0.05 was considered statistically significant. Immunohistochemical quantitative analysis data were made correlation analysis by Origin 8.

## Results

### The Time Course of Pathological, Blood Chemical, and Cytokine Changes in ConA-Induced Hepatitis

**Figure 1:**
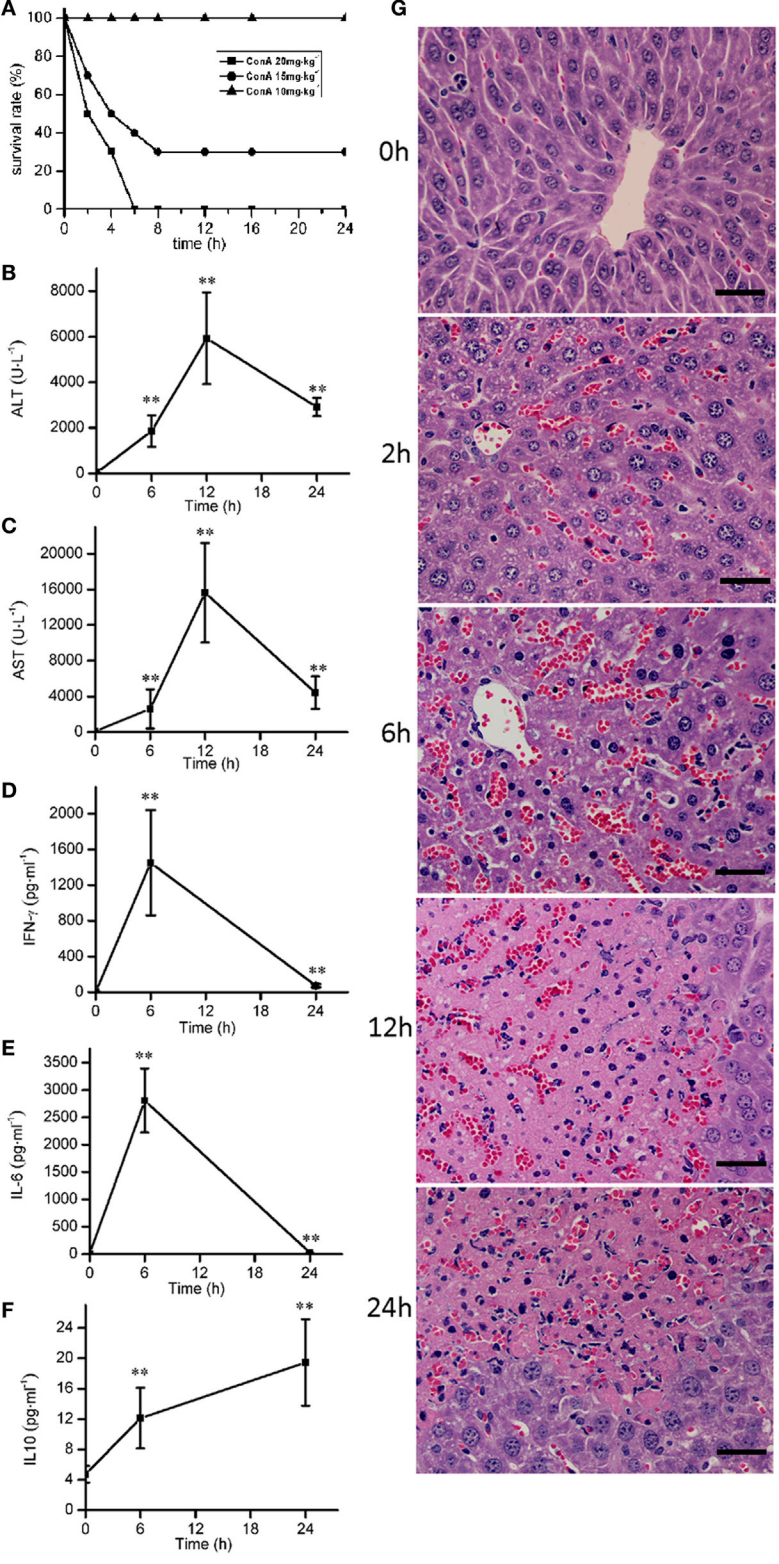
The time course of pathological, blood chemical, and cytokine changes in ConA-induced hepatitis. **(A)** The survival rates were decreased with the increase of ConA. **(B,C)** The serum levels of alanine transaminase, aspartate transaminase after ConA (10 mg⋅kg^−1^) administration. **(D–F)** The changes of serum levels of IFN-γ, IL-6, and IL-10 after ConA (10 mg⋅kg^−1^) administration. **(G)** Representative micrographs depicting H&E stained hepatic sections observed under 40× objectives after ConA (10 mg⋅kg^−1^) administration. Scale bars, 50 µm. Mice were administered with a single intravenous injection of saline containing ConA. The mice treated with intravenous injection of normal saline only were used as control (time 0 h). Error bars indicate mean ± SD. **P < 0.01 compared with control group (*n* = 6).

ConA (10, 15, and 20 mg⋅kg^−1^) were injected intravenously to mice to induce the survival rates of 100, 30, and 0%, respectively (Figure 1A). ConA (10 mg⋅kg^−1^) was selected to monitor the time course of pathological, blood chemical, and cytokine changes in ConA-induced hepatitis. The serum levels of ALT, AST were elevated from 6 h after ConA administration, got peak at 12 h and declined at 24 h (Figures 1B,C). The levels of IL-6 and IFN-γ reached peak at 6 h (Figures 1D,E) and decreased at 24 h. The levels of IL-10 kept the increase from 6 to 24 h (Figure 1F). Histological analysis showed that HE-stained hepatic sections from control group was microscopically normal. At 6 h after ConA challenge, serious congestion occurred in hepatic sinusoid, a great number of inflammatory cells were found in liver tissues, and the hepatocytes cell death appeared with the nuclei condensation. At 12 h, patches of hepatocytes death occurred around the central vein region with the cytoplasm red staining. The lesions were still serious at 24 h (Figure 1G).

### Comparative Proteomic Analysis Between ConA-Induced Hepatitis Mice and Normal Mice

**Figure 2:**
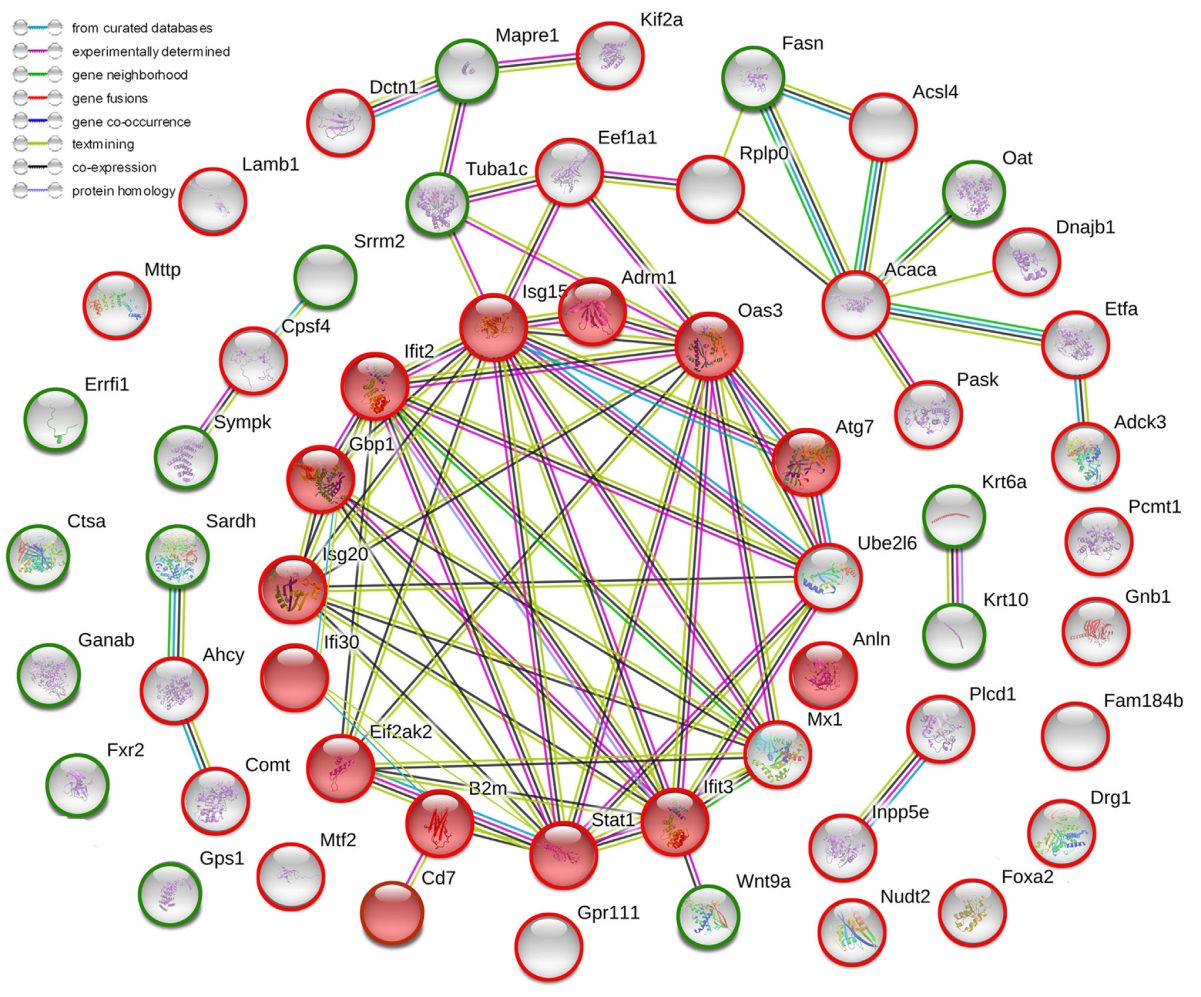
The current interactions of differentially expressed proteins in liver tissues of ConA-induced hepatitis mice compared with that of normal mice. The red nodes represented the functional enrichments of proteins in immune system. The red circles represented upregulated proteins while the green circles represented downregulated proteins. The network was made on the basis of STRING.

After the administration with ConA (10 mg⋅kg^−1^), the proteomic analysis highlighted 57 differentially expressed proteins, including 41 upregulated ones and 16 downregulated ones. They were listed in Table 2. The current interaction network made on the basis of STRING was shown in Figure 2. A large number of proteins showed functional enrichments in immune system processes, including Cd7, B2m, Anln, Oas3, Eif2ak2 (Pkr), Isg15, Gbp1, Ifi30, Ifit2, Ifit3, Isg20, Atg7, Adrm1 and Stat1. The levels of B2m, T-cell antigen Cd7 were upregulated which was associated with the activation and proliferation of T lymphocytes (51, 52). The IFN-induced antiviral ISGs have direct antimicrobial and immunomodulatory effects (53–55). These proteins constructed a complex network which co-induced immune disturbance during the course of hepatitis.

**Table 2:** Protein changes in mice liver following ConA exposure for 12 h.

UniProt ID	Gene name	Protein description	Fold-change
			ConA/Normal saline
E9Q4J9	Adgrf2	Adhesion G-protein coupled receptor F2	16.37
Q8CEE6	Pask	PAS domain-containing serine/threonine-protein kinase	10.94
Q9JKV1	Adrm1	Proteasomal ubiquitin receptor ADRM1	7.91
P50247	Ahcy	Adenosylhomocysteinase	6.93
Q9QZU9	Ube2l6	Ubiquitin/ISG15-conjugating enzyme E2 L6	5.99
P62874	Gnb1	Guanine nucleotide-binding protein G(I)/G(S)/G(T) subunit beta-1	5.33
P50283	Cd7	T-cell antigen CD7	4.75
Q64339	Isg15	Ubiquitin-like protein ISG15	4.66
O08601	Mttp	Microsomal triglyceride transfer protein large subunit	4.57
Q9QUJ7	Acsl4	Long-chain-fatty-acid—CoA ligase 4	4.18
Q8R3B1	Plcd1	1-phosphatidylinositol 4,5-bisphosphate phosphodiesterase delta-1	4.16
Q0KK56	Fam184b	Protein FAM184B	3.62
Q03963	Eif2ak2/Pkr	Interferon-induced, double-stranded RNA-activated protein kinase	3.59
Q8BQZ5	Cpsf4	Cleavage and polyadenylation specificity factor subunit 4	3.54
P10126	Eef1a1	Elongation factor 1-alpha 1	3.53
P56380	Nudt2	Bis(5’-nucleosyl)-tetraphosphatase [asymmetrical]	3.42
P28740	Kif2a	Kinesin-like protein KIF2A	3.35
Q5SWU9	Acaca	Acetyl-CoA carboxylase 1	3.04
Q60936	Adck3	Atypical kinase COQ8A, mitochondrial	2.99
Q9QYJ3	Dnajb1	DnaJ homolog subfamily B member 1	2.89
P42225	Stat1	Signal transducer and activator of transcription 1	2.79
P35583	Foxa2	Hepatocyte nuclear factor 3-beta	2.78
P23506	Pcmt1	Protein-l-isoaspartate (d-aspartate) *O*-methyltransferase	2.76
P32233	Drg1	Developmentally regulated GTP-binding protein 1	2.74
Q8VI93	Oas3	2′-5′-oligoadenylate synthase 3	2.67
Q9D906	Atg7	Autophagy-related protein 7	2.63
Q64112	Ifit2	Interferon-induced protein with tetratricopeptide repeats 2	2.46
Q99LC5	Etfa	Electron transfer flavoprotein subunit alpha	2.45
Q9JII1	Inpp5e	72 kDa inositol polyphosphate 5-phosphatase	2.39
O08788	Dctn1	Dynactin subunit 1	2.35
P01887	B2m	Beta-2-microglobulin	2.34
P02469	Lamb1	Laminin subunit beta-1	2.18
O88587	Comt	Catechol *O*-methyltransferase	2.17
Q8K298	Anln	Anillin	2.13
Q01514	Gbp1	Guanylate-binding protein 1	2.08
P09922	Mx1	Interferon-induced GTP-binding protein Mx1	2.04
P14869	Rplp0	60S acidic ribosomal protein P0	1.98
Q02395	Mtf2	Metal-response element-binding transcription factor 2	1.98
Q9ESY9	Ifi30	Gamma-interferon-inducible lysosomal thiol reductase	1.97
Q9JL16	Isg20	Interferon-stimulated gene 20 kDa protein	1.88
Q64345	Ifit3	Interferon-induced protein with tetratricopeptide repeats 3	1.63
P02535	Krt10	Keratin, type I cytoskeletal 10	0.55
Q8R5M2	Wnt9a	Protein Wnt-9a	0.49
Q9WVR4	Fxr2	Fragile X mental retardation syndrome-related protein 2	0.47
Q99LB7	Sardh	Sarcosine dehydrogenase, mitochondrial	0.44
Q61166	Mapre1	Microtubule-associated protein RP/EB family member 1	0.39
F8WJD4	Sympk	Symplekin	0.39
Q99LD4	Gps1	COP9 signalosome complex subunit 1	0.38
P68373	Tuba1c	Tubulin alpha-1C chain	0.36
Q8BTI8	Srrm2	Serine/arginine repetitive matrix protein 2	0.34
Q8BHN3	Ganab	Neutral alpha-glucosidase AB	0.33
P19096	Fasn	Fatty acid synthase	0.29
P16675	Ctsa	Lysosomal protective protein	0.23
P01845	Iglc3	Ig lambda-3 chain C region	0.21
P29758	Oat	Ornithine aminotransferase, mitochondrial	0.18
Q99JZ7	Errfi1	ERBB receptor feedback inhibitor 1	0.09
P50446	Krt6a	Keratin, type II cytoskeletal 6A	0.08

*Table showed the mean fold changes of proteins in model mice versus normal mice with *p* values less than 0.05 (*n* = 3), where ratios >1.25 were upregulation; ratios <0.8 were downregulation*.

The autophagy and UPS are two highly conserved degradative pathways that regulate protein homeostasis by the clearance of cytoplasmic materials in eukaryotic cells. Here, the proteomic analysis revealed that they might be disturbed by a variety of IFN-induced proteins. For example, the highly elevated Isg15 has been shown to antagonize the ubiquitin/proteasome pathway. The protein ISGylation interferes with ubiquitination *via* substrate competition at E2/E3 level (56). Ube2l6 (UbcH8) is a major E2 enzyme for Isg 15 conjugation (57). Here, it was also upregulated. The Isg15 conjugation pathway overlapped the ubiquitin conjugation pathway, leading to changes in degradation of cellular protein.

The impaired of the proteolytic capacity of the UPS can promote inflammation or induce cell death in autoinflammation (58). Here, the dysregulation of proteasome capacity was revealed by the upregulation of Adrm1. Adrm1 (also named Rpn13 or Arm-1) was previously identified as an adhesion-regulating molecule of T cells (59). Adrm1 has also been described as an IFN-γ-inducible, heavily glycosylated membrane protein of 110 kDa (Gp110) (60). Adrm1 (Rpn13 in yeast) is a subunit of 19S-regulatory particle (19S-RP). The 26S proteasome is a large, multi-protein consisting of the 20S catalytic core particles (20S CP) and 19S-RP. Adrm1 is a novel ubiquitin receptor, it is able to recruit ubiquitinated substrates to the 19S-RP, and then fed them into the proteasome’s catalytic 20S-CP for degradation (61, 62). However, the overproduction of Adrm1 reduced the degradation of the most short-lived proteins, and the transfection of the C-terminal half of Adrm1 slowed proteolysis and induced cell death (63). The reason might be that Adrm1 was also the proteasomal receptor for Uch37, and it could recruit and activate Uch37, a deubiqitinating enzyme (63, 64). The Uch37 disassemble the poly-Ub chain and recycle the ubiquitin during proteasomal degradation (65). Interaction of Adrm1 with Uch37 increased the production of triubiquitin (Ub3), diubiquitin (Ub2), and monoubiquitin (Ub1) (66). Uch37 was activated by Adrm1 to form Uch37–Adrm1 complex, exhibiting 12-fold higher activity than Uch37 alone (67).

Multilple ubiquitins were released from changed degradation when the ubiquitin binding sites were occupied by Isg15. It was proposed that Adrm1 and Isg15 might work together to induce the overexpression of Ub in ConA-induced hepatitis. The p62 would undergo ubiquitylation under Ub stress and activate selective autophagy (68). The p62-dependent autophagic clearance of accumulated polyubiquitinated proteins is a compensatory process for the loss of proteasome ability (69). In addition, the Isg15 and ISGylation would lead to upregulation of p62 and LC3II, then the Isg15-congugated proteins were also degraded by lysosome through the conjugation with p62 (70).

Double-stranded RNA-dependent protein kinase (Pkr) is an IFN-inducible anti-viral protein. Pkr could participate in the inflammation and immune regulation by not only pro-apoptotic activity but also pro-autophagic activity (71). It is also known as eukaryotic translation initiation factor 2-alpha kinase 2 (eIF2α). EIF2α mediated the activation of the microtubule-associated protein LC3 (72). Atg7 is required by the induction of mammalian autophagy, which is one E1 enzyme for the activation of autophagy-essential ubiquitin-like protein LC3 (73). Thus, the upregulation of Pkr and Atg7 might lead to the increased expression of LC3II.

In summary, the overexpression of p62 would be associated with the accumulation of ISGylated proteins and autophagosomes. The ubiquitylation of p62 could liberate it to recognize polyubiquitylated cargoes for autophagy (68). It engulfed the aggregates in autophagosomes and delivered them to lysosome for degradation. The overexpression of p62 represented the accumulated autophagosome and a blockage of autophagic flux, eventually leading to autophagic cell death (21).

### Validation of the Proteomic Analysis by Immunohistochemistry

**Figure 3:**
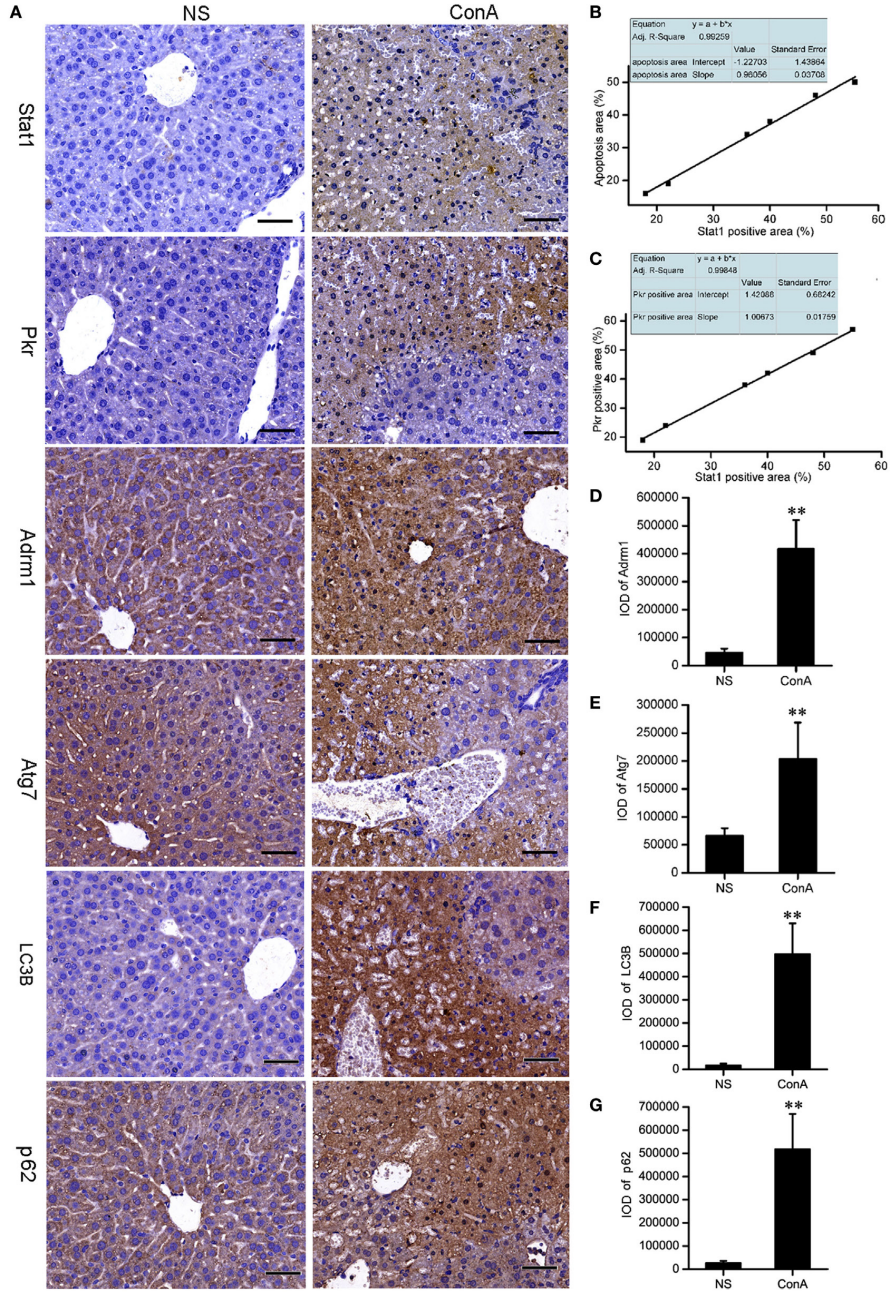
The validation of the proteomic data of Stat1, Pkr, Atg7, and Adrm1 by IHC. The LC3B and p62 were detected by IHC. **(A)** The IHC of Stat1, Pkr, Atg7, Adrm1, LC3B, and p62 of control mice and model mice. Positive correlations of Stat1 with apoptosis **(B)**, Stat1 with Pkr **(C)** in the sections of ConA treatment group were shown. Integrated optical density of Adrm1 **(D)**, Atg7 **(E)**, LC3B **(F)**, and p62 **(G)** showed that they were upregulated after ConA treatment. **p < 0.01 compared with the control group (*n* = 6).

It was proposed that LC3 and p62 might accumulate in the liver tissue after ConA challenge. Recent reports showed that IHC staining for LC3B and p62 might be the best approach to monitor autophagy in large human tissue sections, when electron microscopy is not feasible (74, 75). We performed IHC staining to validate them. The expressions of Stat1 and Pkr in control group were rarely seen, but they were increased sharply in model group and expressed at the apoptotic or necrotic areas at 12 h after ConA challenge. Correlation analysis showed that Stat1 expression was positively correlated with the apoptosis, and Pkr expression was positively correlated with Stat1 expression. Adrm1, Atg7, LC3B, and p62 were expressed in the cytoplasm of normal hepatocytes. They were overexpressed in patchy apoptotic areas together with Stat1 after ConA challenge. The overexpressions of LC3B and p62 were also verified by IHC analysis. The IOD of IPP were representative parameters to assess the immunostaining quantification, and provided a more accurate analysis of protein expression (76), so the IOD provided by IPP was performed to assess the IHC quantification here. The IHC results of Stat1, Pkr, Adrm1, and Atg7 were consistent with our proteomic analysis (Figure 3).

### ARC Pretreatment Attenuated the ConA-Induced Hepatitis

**Figure 4:**
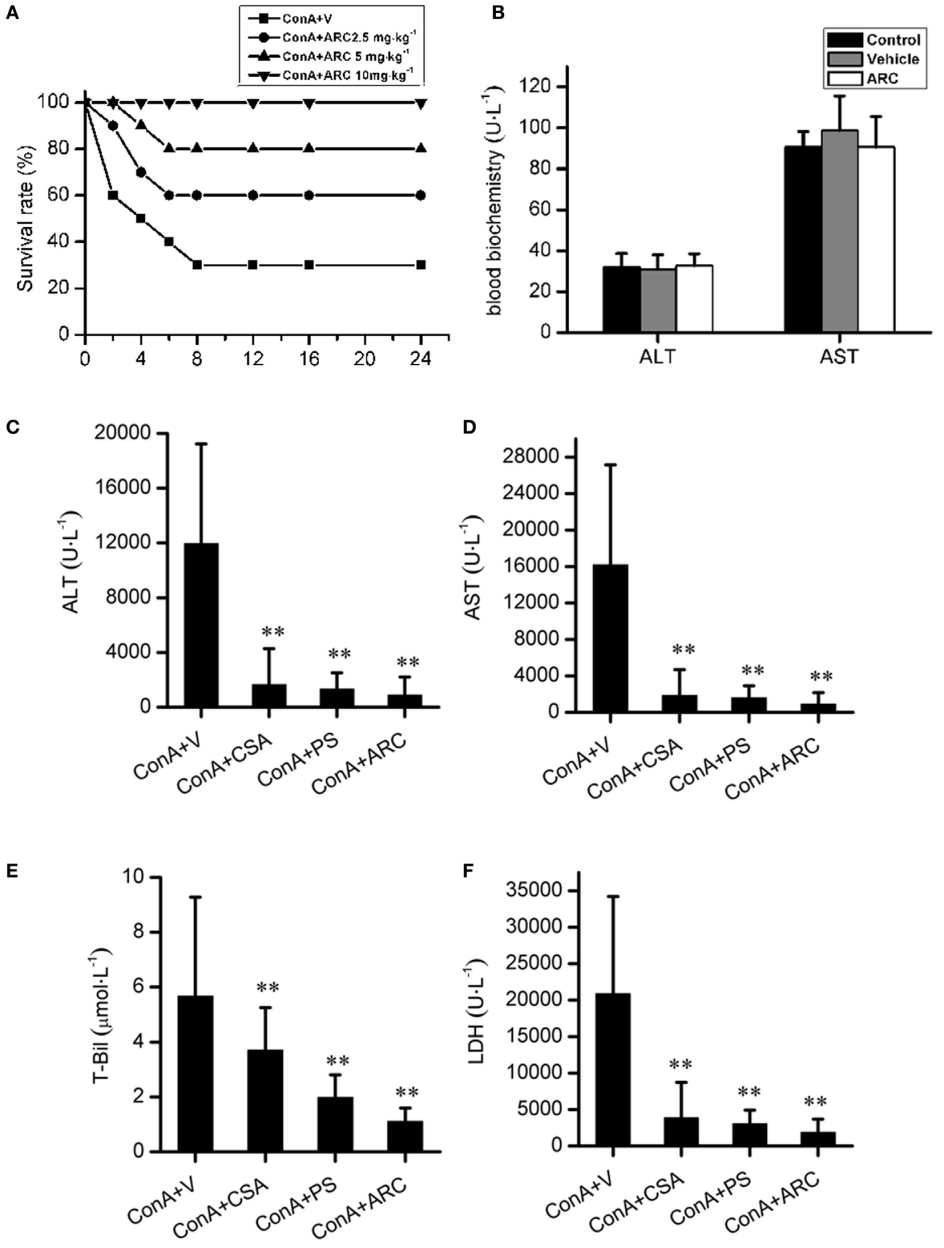
The protective effects of ARC on survival rates and liver function in ConA-induced hepatitis. ARC (2.5, 5, and 10 mg⋅kg^−1^) pretreatment significantly increased survival rates after ConA (15 mg⋅kg^−1^) administration **(A)**. In the presence of drugs or vehicle treatments twice per day for 10 days, the blood biochemical measurements showed that ARC or vehicle alone had no effects on normal liver functions **(B)**. CSA, PS, and ARC markedly decreased the serum levels of alanine transaminase, aspartate transaminase, TBIL, and lactate dehydrogenase at 12 h after ConA (10 mg⋅kg^−1^) administration **(C–F)**. **p < 0.01 compared with model group (*n* = 8). The mice pre-treated with vehicle were used as model group.

**Figure 5:**
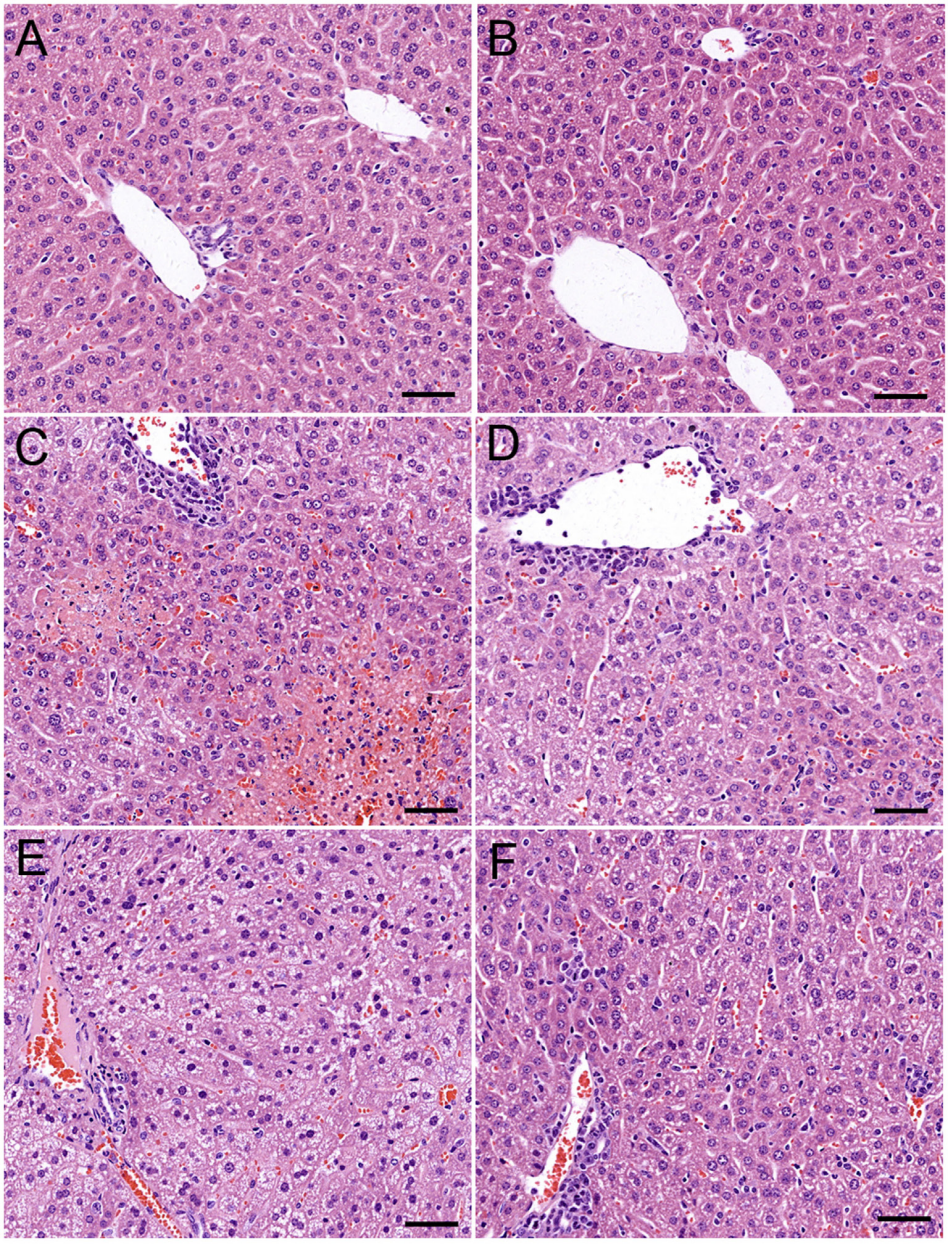
The protective effects of ARC on liver pathology of ConA-induced hepatitis mice. ARC or vehicle alone had no effects on morphology of liver section **(A,B)**. Serious liver damage was found in model group at 12 h after ConA (10 mg⋅kg^−1^) administration **(C)**. Portal inflammation, hepatocytes edema, severe congestion in hepatic sinusoid around central veins, and focal or confluent necrosis occurred significantly. ARC significantly inhibited ConA-induced histological changes, which was comparable with effects of CSA and PS **(D–F)**.

ARC has distinct anti-inflammatory property. We proposed that it might extent protective effects on immune-mediated hepatitis. The protective effects of ARC pretreatment on ConA-induced hepatitis were tested in mice. Intravenous injection with ConA (15 mg⋅kg^−1^) led to a survival rate of 30% in mice. ARC (2.5, 5, and 10 mg⋅kg^−1^) pretreatment significantly reversed the survival rates, when there were no deaths in ARC (10 mg⋅kg^−1^) group (Figure 4A). ConA (10 mg⋅kg^−1^) was selected to investigate the protective mechanism of ARC on ConA-induced hepatitis. CSA and PS have been confirmed to be effective in the treatment of the AIH (77), so they were selected as positive parallel controls. The ARC or vehicle alone had no effects on normal liver functions (Figure 4B). CSA, PS, and ARC markedly decreased the serum levels of ALT, AST, TBIL, and LDH at 12 h after ConA administration (Figures 4C–F). Pathological sections showed that ARC or vehicle alone had no effects on morphology of liver section (Figures 5A,B). Serious liver damages were found in model group at 12 h after ConA (10 mg⋅kg^−1^) administration (Figure 5C), including portal inflammation, hepatocytes edema, severe congestion in hepatic sinusoid around central veins, and focal or confluent necrosis. ARC significantly inhibited ConA-induced histological changes, which was comparable with effects of CSA and PS (Figures 5D–F). The scores of liver injury for each mouse were histopathologically evaluated using a semiquantitative scoring method revised previously described (78, 79). Liver injury was graded from 0 (normal) to 4 (severe) in four categories: inflammatory cell infiltration, congestion, edema, and necrosis. The total liver injury score (TLIS) was calculated by adding the individual scores for each category. The results showed that CSA, PS, and ARC all significantly alleviated ConA-induced hepatitis (Table 3). The TLIS was lowest in ARC pretreatment group. In PS treatment group, the inflammation was lightest, but the hepatocytes edema was rather obvious which might be attributed to the side effects of glucocorticoids.

**Table 3:** Components and total values of liver injury score.

	Control	ConA + V	ConA + CsA	ConA + PS	ConA + ARC
Portal inflammation	0 ± 0	2.0 ± 0.89^##^	1.4 ± 0.48*	0.5 ± 0.65**	1.0 ± 0.64**
Edema	0.1 ± 0.33	2.8 ± 0.89^##^	1.4 ± 0.48**	0.8 ± 0.55**	0.5 ± 0.58**
Congestion	0.3 ± 0.43	2.3 ± 0.71^##^	1.3 ± 0.43**	2.4 ± 0.85	0.4 ± 0.97**
Necrosis	0 ± 0	2.5 ± 1.20^##^	0.4 ± 0.48**	0.3 ± 0.44**	0.3 ± 0.42**
Total liver injury score	0.4 ± 0.48	9.5 ± 2.20^##^	4.38 ± 0.70**	3.9 ± 1.23**	2.1 ± 0.54**

Data are expressed as mean ± SD (*n* = 8), ^##^*p <* 0.01 compared with control; *P < 0.05 compared with model mice treated with vehicle; **P < 0.01 compared with model mice treated with vehicle.

### ARC Impaired the Levels of Inflammatory Cytokines in ConA-Induced Hepatitis

The main role of IL-10 is to limit inflammatory responses and regulate activation of several immune cells (80). Systemic recombinant IL-10 administration is feasible as one therapy for AIH (77). ConA-induced hepatitis in mice is prevented by exogenous IL-10 and exacerbated by anti-IL-10 mAb or IL-10 KO (81). IL-6 exerts various effects on hepatocytes and lymphocytes in acute or chronic inflammatory diseases and anti-IL-6 receptor antibody was developed to treat arthritis and other refractory immune-mediated diseases (82). Thus, we selected IL-10 and IL-6 as well as IFN-γ to test the anti-inflammatory properties of ARC.

**Figure 6:**
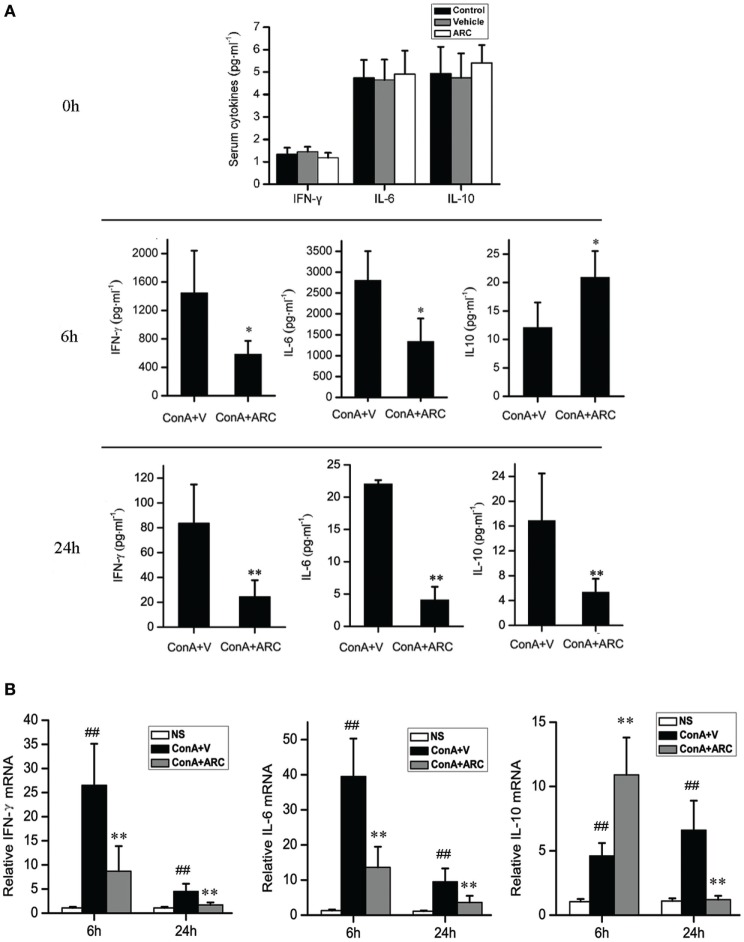
The influence of ARC on the cytokines in serum and liver tissues after ConA (10 mg⋅kg^−1^) administration with and without ARC pretreatment. **(A)** The influence of ARC on the release of cytokines. In the presence of drugs or vehicle treatments twice per day for 10 days, ARC or vehicle alone had no effects on the inflammatory responses in normal mice. The levels of IL-6 and IFN-γ were decreased in serum in ARC pretreatment group at 6 and 24 h, and the level of IL-10 was still kept increasing from 6 to 24 h at model group, ARC pretreatment further increased the level of IL-10 at 6 h, but the level of IL-10 in ARC pretreatment mice was lower than that in model mice at 24 h. **(B)** The influence of ARC on the mRNA level of cytokines in liver tissues. The mRNA levels of IL-6 and IFN-γ in liver tissues were both increased after ConA administration. They were decreased by ARC pretreatment at 6 and 24 h. ARC pretreatment further increased the mRNA level of IL-10 in liver tissue at 6 h after ConA administration. At 24 h, the mRNA level of IL-10 was further increased in model group, but it returned to normal level in ARC pretreatment group. ^##^*p* < 0.01 compared with normal group; *p < 0.05, **p < 0.01 compared with model group (*n* = 8). The mice pre-treated with vehicle were used as model group.

The level of IL-10 was elevated along with the progression of inflammation after ConA administration, which might be a feedback to suppress liver inflammation. ARC pretreatment further increased the mRNA level of IL-10 in liver tissue and the release of IL-10 to serum at 6 h after ConA administration. The levels of IL-10 were decreased at 24 h, which represented a resolution of inflammation in ARC pretreatment group at this time point. The serum levels of IL-6 and IFN-γ were shown in Figure 6A. ARC pretreatment markedly decreased the levels of IL-6 and IFN-γ at 6 and 24 h. The mRNA levels of IL-6 and IFN-γ in liver tissues were both elevated after ConA administration, but they were reversed by ARC pretreatment at 6 and 24 h (Figure 6B). The data demonstrated that ARC effectively suppressed the levels of pro-inflammatory cytokines, while it induced the ones of anti-inflammatory cytokines.

### Comparative Proteomic Analysis Between ARC Pre-Treated and Vehicle Pre-Treated Mice Models

**Figure 7:**
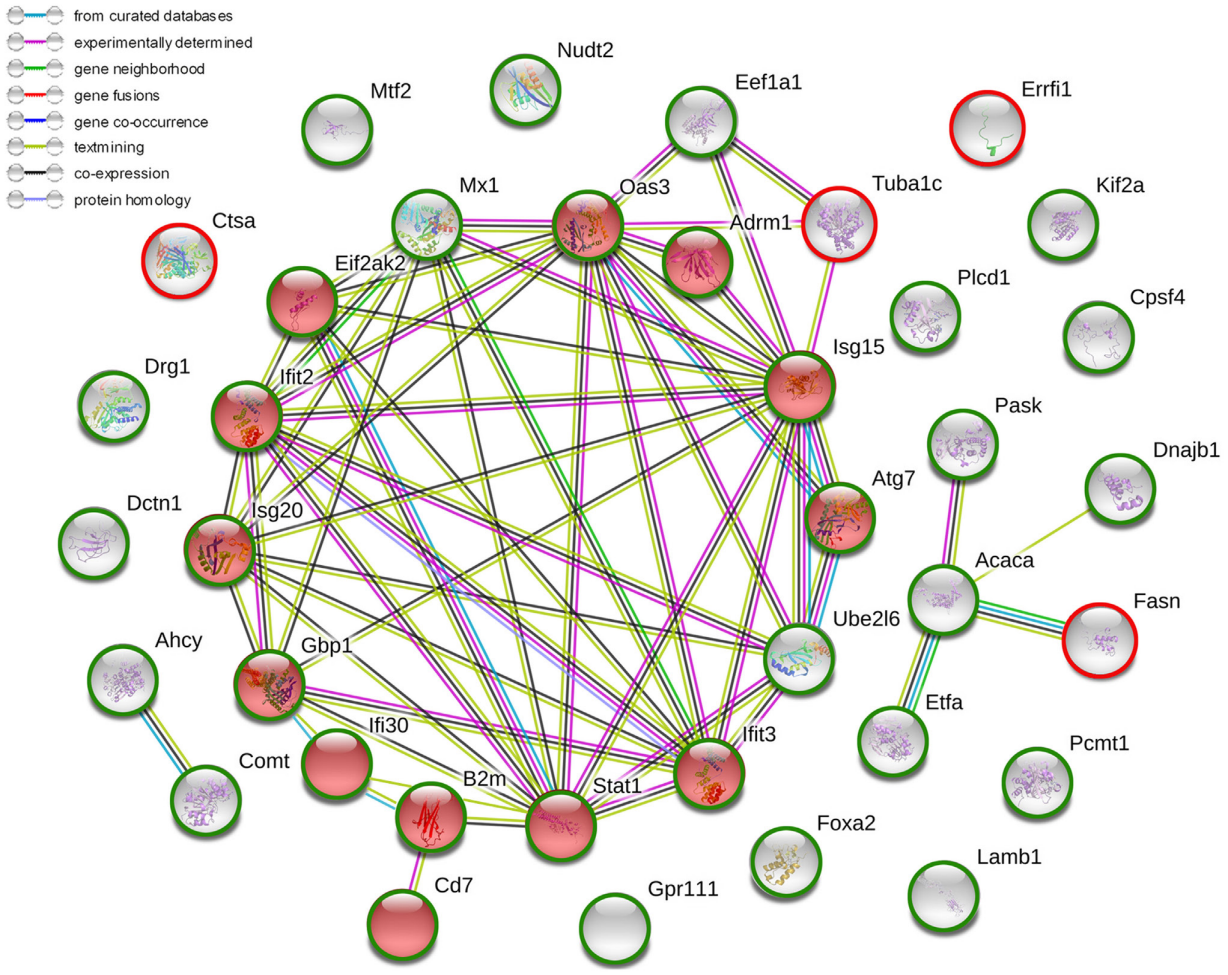
The current interactions of changed proteins in liver tissues with ARC pretreatment compared with those with vehicle pretreatment at 12 h after ConA administration. The red nodes represented the functional enrichments of proteins in immune system. The red circles represented upregulated proteins while the green circles represented downregulated proteins. The network was made on the basis of STRING.

The proteomic analysis was carried out to investigate the effects of ARC on ConA-induced hepatitis. Compared to vehicle pre-treated group, 37 changed proteins were highlighted in ARC pre-treated group, including 5 upregulated ones and 32 downregulated ones (Table 4). The STRING analysis showed that the proteins enriched in immune system were downregulated in ARC pre-treated group, including Cd7, B2m, Oas3, Eif2ak2 (Pkr), Isg15, Gbp1, Ifi30, Ifit2, Ifit3, Isg20, Atg7, Adrm1, and Stat1 (Figure 7). It indicated that the activation of immune system and autophagy were suppressed by ARC pretreatment.

**Table 4:** Protein changes in mice liver tissues with or without ARC pretreatments following ConA exposure for 12 h.

UniProt ID	Gene name	Protein description	Fold-change
			ConA + ARC/ConA + vehicle
E9Q4J9	Adgrf2	Adhesion G-protein coupled receptor F2	0.09
Q8CEE6	Pask	PAS domain-containing serine/threonine-protein kinase	0.13
Q9JKV1	Adrm1	Proteasomal ubiquitin receptor ADRM1	0.18
P50247	Ahcy	Adenosylhomocysteinase	0.21
Q9QZU9	Ube2l6	Ubiquitin/ISG15-conjugating enzyme E2 L6	0.23
P50283	Cd7	T-cell antigen CD7	0.29
Q64339	Isg15	Ubiquitin-like protein ISG15	0.32
Q9QUJ7	Acsl4	Long-chain-fatty-acid—CoA ligase 4	0.33
Q8R3B1	Plcd1	1-phosphatidylinositol 4,5-bisphosphate phosphodiesterase delta-1	0.34
Q03963	Eif2ak2/Pkr	Interferon-induced, double-stranded RNA-activated protein kinase	0.39
P10126	Eef1a1	Elongation factor 1-alpha 1	0.43
P56380	Nudt2	Bis(5′-nucleosyl)-tetraphosphatase [asymmetrical]	0.41
P28740	Kif2a	Kinesin-like protein KIF2A	0.42
Q5SWU9	Acaca	Acetyl-CoA carboxylase 1	0.46
Q9QYJ3	Dnajb1	DnaJ homolog subfamily B member 1	0.48
P42225	Stat1	Signal transducer and activator of transcription 1	0.57
P35583	Foxa2	Hepatocyte nuclear factor 3-beta	0.55
P23506	Pcmt1	Protein-l-isoaspartate (d-aspartate) *O*-methyltransferase	0.51
P32233	Drg1	Developmentally-regulated GTP-binding protein 1	0.51
Q8VI93	Oas3	2′-5′-oligoadenylate synthase 3	0.52
Q9D906	Atg7	Autophagy-related protein 7	0.53
Q64112	Ifit2	Interferon-induced protein with tetratricopeptide repeats 2	0.57
Q99LC5	Etfa	Electron transfer flavoprotein subunit alpha	0.57
O08788	Dctn1	Dynactin subunit 1	0.61
P01887	B2m	Beta-2-microglobulin	0.64
P02469	Lamb1	Laminin subunit beta-1	0.64
O88587	Comt	Catechol *O*-methyltransferase	0.65
Q01514	Gbp1	Guanylate-binding protein 1	0.67
P09922	Mx1	Interferon-induced GTP-binding protein Mx1	0.69
Q02395	Mtf2	Metal-response element-binding transcription factor 2	0.71
Q9ESY9	Ifi30	Gamma-interferon-inducible lysosomal thiol reductase	0.71
Q9JL16	Isg20	Interferon-stimulated gene 20 kDa protein	0.74
Q64345	Ifit3	Interferon-induced protein with tetratricopeptide repeats 3	0.76
Q99LD4	Gps1	COP9 signalosome complex subunit 1	1.88
P68373	Tuba1c	Tubulin alpha-1C chain	1.98
Q8BHN3	Ganab	Neutral alpha-glucosidase AB	2.16
P19096	Fasn	Fatty acid synthase	2.46
P16675	CtsA	Lysosomal protective protein	3.11
P01845	Iglc3	Ig lambda-3 chain C region	3.41
Q99JZ7	Errfi1	ERBB receptor feedback inhibitor 1	7.94

*Table showed the mean fold changes of proteins in ARC pre-treated model group verses vehicle group with *p* values less than 0.05 (*n* = 3), where ratios >1.25 were upregulation; ratios <0.8 were downregulation*.

### ARC Pretreatment Alleviated the Hepatocyte Apoptosis and Autophagy in ConA-Induced Hepatitis

**Figure 8:**
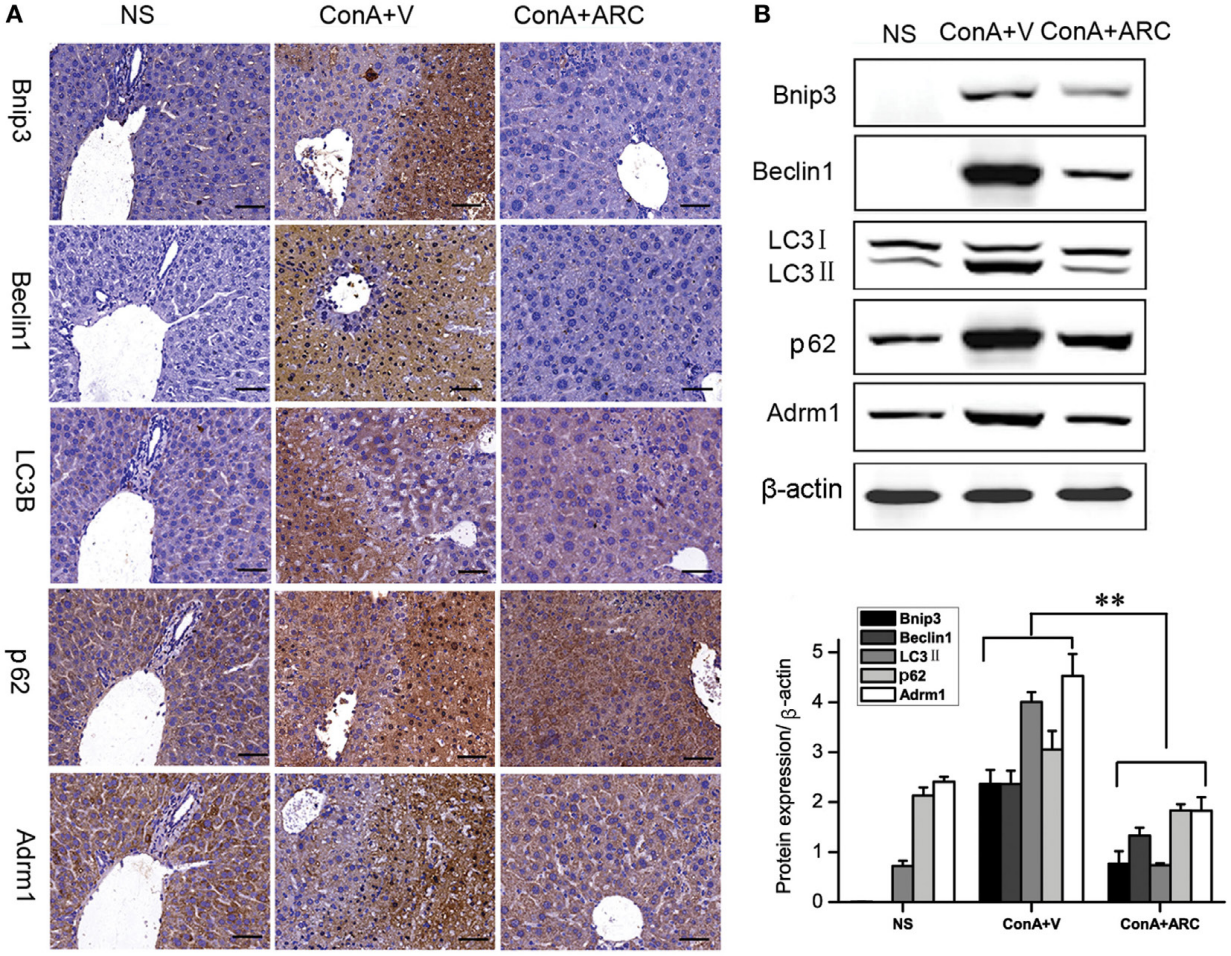
ARC downregulated apoptosis and autophagy in ConA-induced hepatitis. **(A)** The expressions of Bnip3, Beclin1, LC3B, p62, and Adrm1 were measured by IHC. **(B)** The protein levels of Bnip3, Beclin1, LC3B, p62, and Adrm1 were measured by Western blot. The values are represented as mean ± SD. **p < 0.01 compared with model group (*n* = 8). The mice pre-treated with vehicle were used as model group.

The expression of Bnip3 (BCL2/adenovirus E1B 19 kDa interacting protein 3) was detected to evaluate the apoptosis. The Beclin1 represented the initiation of autophagy (83). Previous studies (23–25, 29) showed that overexpressions of Bnip3 and Beclin1 were involved in ConA-induced hepatitis. Present IHC analysis showed that Bnip3 and Beclin 1 were hardly seen in normal mice, and they were overexpressed in the apoptosis/necrosis regions. Previous proteomic analysis revealed the upregulation of Adrm1 mediated dysregulation of proteasome capacity and then influence autophagy, here WB and IHC results confirmed that Adrm1 was upregulated and overexpressed with LC3II and p62 in apoptotic/necrotic areas. All of their expressions were significantly reversed in ARC pre-treated mice (Figure 8).

**Figure 9:**
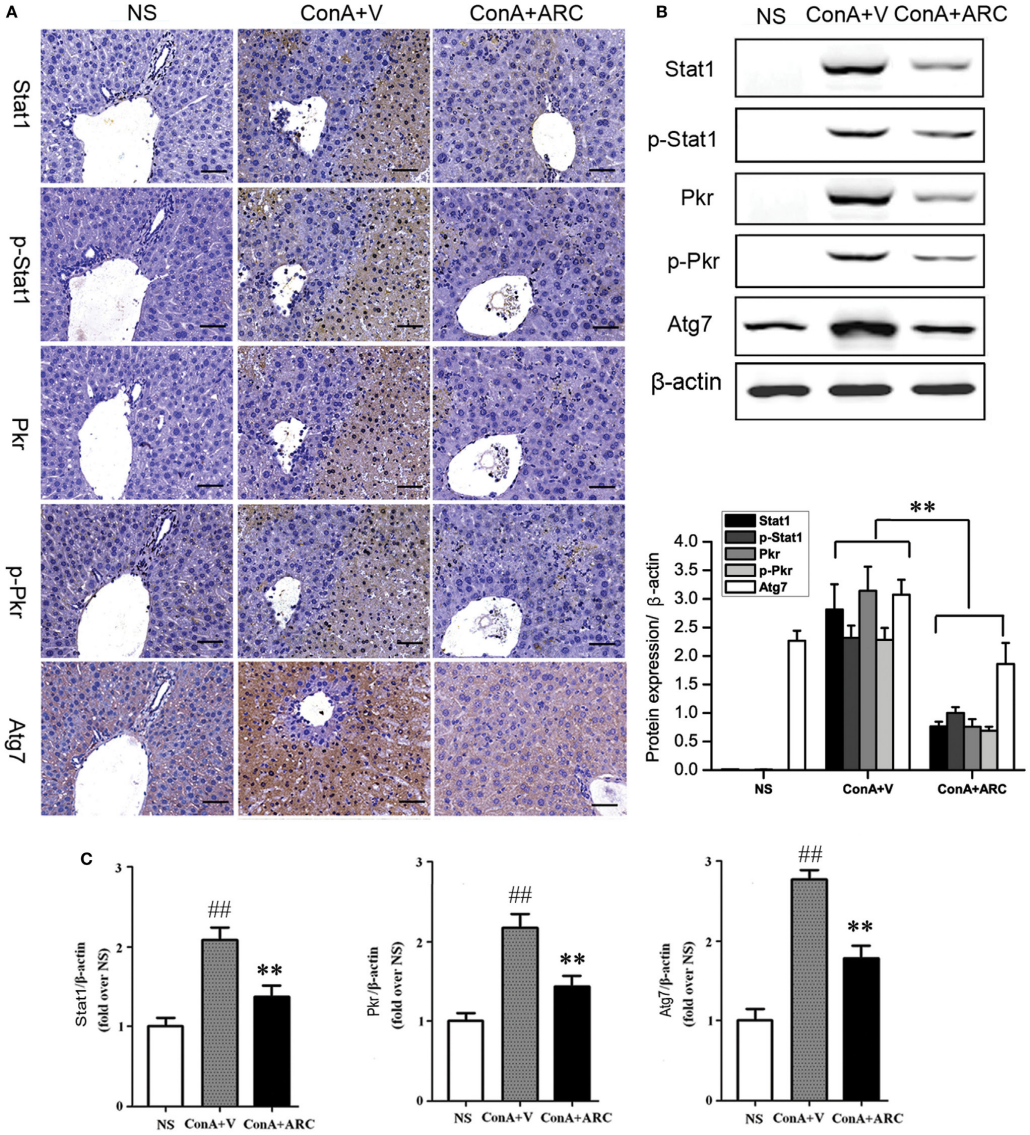
ARC inhibited the expressions of Stat1, p-Stat1, Pkr, p-Pkr, and Atg7. **(A)** The expressions of Stat1, p-Stat1, Pkr, p-Pkr, and Atg7 were measured by IHC. **(B)** The protein levels of Stat1, p-Stat1, Pkr, p-Pkr, and Atg7 were measured by Western blot. **(C)** The mRNA level of Stat1, Pkr, and Atg7 were measured by RT-PCR. The values are represented as mean ± SD. ^##^*p* < 0.01 compared with normal group; **p < 0.01 compared with model group (*n* = 8). The mice pre-treated with vehicle were used as model group.

Present proteomic analysis demonstrated that IFN-γ/Stat1 signaling played a major role in the activation of immune system. To verify the possible mechanisms hidden behind ARC pretreatment, we investigated the protective effects of ARC on the synthesis and activation of Stat1 in liver tissues. As an important factor involved in inflammation, apoptosis, and autophagy, Pkr was studied. The effect of ARC on the synthesis of Atg7 was also investigated. The results showed that Stat1, p-Stat1, Pkr, and p-Pkr were barely expressed in normal liver and Atg7 was weakly positive in liver section of normal mice. However, they were all overexpressed at apoptotic regions. Their expressions were decreased in ARC pre-treated mice (Figures 9A,B). Likewise, the mRNA levels of Stat1, Pkr, and Atg7 in liver tissues were all increased after ConA administration. They were reversed by ARC pretreatment (Figure 9C). All these data suggested that ARC indeed inhibited the autophagy and apoptosis in ConA-induced hepatitis.

## Discussion

The liver is not only a critical metabolic organ but also a lymphoid organ with unique immunological properties (84). Different phenotypic and functional lymphoid cells were discovered in liver that contribute to immune surveillance against toxins, pathogens, tumor cells and self-antigens of the liver (85). A peripheral break of tolerance against liver-expressed antigens is sufficient to induce an immune liver disease. Meanwhile, T-cell trapping plays an important role in the initiation and perpetuation of immune hepatitis, which can induce the expressions of specific chemokines and adhesion molecules (86). Animal studies showed that ConA-induced hepatitis model was extremely suitable to mimic immune hepatitis. Here, the proteomic data on ConA-induced hepatitis model displayed a comprehensive view for the abnormal immune system and provided some new evidences for the ConA-induced hepatitis model to mimic autoimmune liver disease and virus hepatitis.

The upregulation of large amounts of IFN-induced proteins represented the activation of immune system in ConA-induced hepatitis. Although these proteins have direct antimicrobial effects (53–55), they also disrupt the immune system and induce hepatitis which is confirmed in present study. They not only cause inflammation and trigger apoptosis, but also affect cell function by inducing autophagy, such as Pkr. Pkr has multifaceted roles in inflammation and immune dysfunction. It interacted with inflammasome components by autophosphorylation (87). It was essential for the LPS-induced activation of Stat1 inflammatory signaling (88). Pkr mediated the IFN-γ-induced injurious effects through STAT1/IRF-1-dependent cell death signaling (89). The inhibition of Pkr stabilized Bax, in which stabilized Bax could not insert into the outer mitochondrial membrane and initiate stress-induced apoptosis (90). Pkr is essential for autophagy induced by herpes simplex virus infection (72), Moreover, Pkr participated in stress-related damages through generation of stress granules (91). Here, the overproduction and activation of Pkr contributed to the apoptosis and autophagy in ConA-induced hepatitis. ARC pretreatment impeded both the expression and activation of Pkr which was beneficial for restriction of liver injury.

As reported, Jak1–Stat1 signaling played an absolutely necessary role in IFN-α induces autophagy in the pathogenesis of autoimmune disease, and many autophagy-related proteins were upregulated in this process (92). In present study, Atg7 is one of upregulated proteins which showed functional enrichments in immune system process. The proteomic analysis showed the upregulation of Atg7 was along with the Stat1, and IHC results demonstrated the overexpression of Atg7 was distributed at apoptotic areas with the locations of Stat1 and p-Stat1. It was suggested that the Stat1 might also account for the upregulation of Atg7. Atg7 is a key pro-autophagic promoting gene which has a critical role in membrane elongation (73). In addition to its important role in autophagy, it also participated in the inflammation and apoptosis. Atg7 participated in the IFN-γ-induced recruitment of the immunity-related GTPases and guanylate-binding proteins (GBPs) to the intracellular pathogen (93), and GBPs could enable rapid activation of inflammasomes in infected macrophages (55). Atg7 deficiency decreased iNOS activity by downregulating Jak2/Stat1α signaling (94), and hyper-activated Atg7 could induce the cell death by modulating p53 activity (95). It was suggested that Atg7 might be the target of immune-related liver disease. The mRNA and protein levels of Atg7 were both decreased in ARC pretreatment group, thus indicating that ARC might protect liver from ConA-induced hepatitis through the inhibition of the upregulation of Atg7.

Among those upregulated proteins enriched in immune system, we speculated that the upregulated Adrm1, Isg15, and Ube2l6 contributed to the imbalance of autophagy and UPS in immune hepatitis. Considering their downregulations in response to ARC pretreatment, they may be the targets to treat immune hepatitis, but the correlation of Adrm1with Stat1 need further investigations. Adrm1, ubiquitin stress, ISGylation proteins, and LC3II jointly led to the overexpression of p62. The overexpression of LC3II and p62 were inhibited by ARC pretreatment. Present study suggested the inhibition of the accumulation of autophagosome by ARC might be due to the suppression of the activated immune system.

During ConA-induced hepatitis, IFN-γ not only induced IFN-induced proteins but also influenced the production of cytokines from other immune cells. As reported, IFN-γ could enhance IL-6 production in activated macrophages (96), and IFN-γ could stimulate the polarization of macrophages into pro-inflammatory M1 macrophages (97). As resident macrophages in liver, KCs can express pro-inflammatory M1 phenotype and the anti-inflammation M2 phenotype according to the immune and metabolic environment (98), and KC was reported to contribute to ConA-induced hepatitis through a Th1 type response-dependent pathway (99). IL-6 is one of indicators of M1-polarized KCs or macrophages (100). It was reported that IL-6 produced by KCs played a significant role in the pathogenesis of ConA-induced hepatitis (101). Further, IL-6 played a key role in CD4^+^ T cell memory formation and contributed to the proliferation and survival of CD4^+^ T cells (102, 103). KCs were crucial for IL-10 production in ConA or HBV tolerance (104, 105), and M2 cells could promote M1 death by IL10 releasing which was beneficial for inhibiting inflammation and hepatocyte injury (106). Previous studies showed that IL-10 not only inhibited Th1 cell producing IFN-γ but also inhibited the production of IL-6 by activated macrophages (96, 107). Previous studies demonstrated that ARC could inhibit IL-2 and IFN gene expression in T lymphocytes, it can also promote the transition of M1-like macrophages into M2-like macrophages (41, 108). The above findings suggested that the protective effects of ARC on ConA-induced hepatitis might be due to the promotion of IL-10 generation and the inhibition of IL-6 and IFN-γ production.

Stat1 was essential for M1 macrophages activation by IFN-γ (97), it also participated IL-6 induced CD4^+^ T cell differentiation into follicular helper cells (Tfh) (109). Tfh cells were reported to correlate with autoantibody production in human autoimmune diseases due to its role in supporting the formation and differentiation of B cells into memory and plasma cells (110). A recent study showed that IL-6 mediated the IFN-α-induced cell apoptosis *via* the activation of Stat1 (111). Both IL-6 and IFN-γ were required for murine mercury-induced autoimmunity (112). They both interplayed with interferon regulatory factor 1 to exacerbate the inflammatory responses. The expressions of Stat1 and p-Stat1 were both decreased in ARC pre-treated mice. It was concluded that ARC alleviated cell autophagy and apoptosis *via* the inhibition of IFN-γ/IL-6/Stat1 signaling in ConA-induced hepatitis. Further, the mRNA level of Stat1 was decreased by ARC pretreatment, thus suggesting that ARC attenuated the cell apoptosis and autophagy in ConA-induced hepatitis by suppressing the synthesis and activation of Stat1.

IL-6 was also involved in the induction of BNIP3 through the activation of Jak/Stat3 signaling (113, 114), and the suppression of IL-6/Jaks/Stat3 signaling might contribute to the inhibition of Bnip3-mediated apoptosis and autophagy in ConA-induced hepatitis (29). Bnip3 is a pro-apoptotic BH3-only protein that mediates mitochondrial dysfunction and cell death *via* the heterodimerization with Bcl-2/Bcl-X(L) and the activations of Bak or Bax (115, 116). Bnip3 mediated the crosstalk between autophagy and apoptosis through the interactions with B-cell-lymphoma (Bcl2) (117). Bcl2 is an anti-apoptotic protein, and stabilized Bcl2 can inhibit ROS-induced apoptosis (118). Bcl2 was also able to inhibit cell autophagy through interaction with Beclin 1 and prevent cell death from elevated level autophagy (119). Bnip3 can disrupt the interaction between Bcl-2 and Beclin 1 to enhance the formation of autophagosome (120). The overexpression of Beclin1 increased the convention of LC3, and enhanced the cisplatin-induced apoptosis (121). ARC decreased apoptosis and autophagy in ConA-induced hepatitis might also by the inhibition of IL6/Bnip3 pathway.

**Figure 10:**
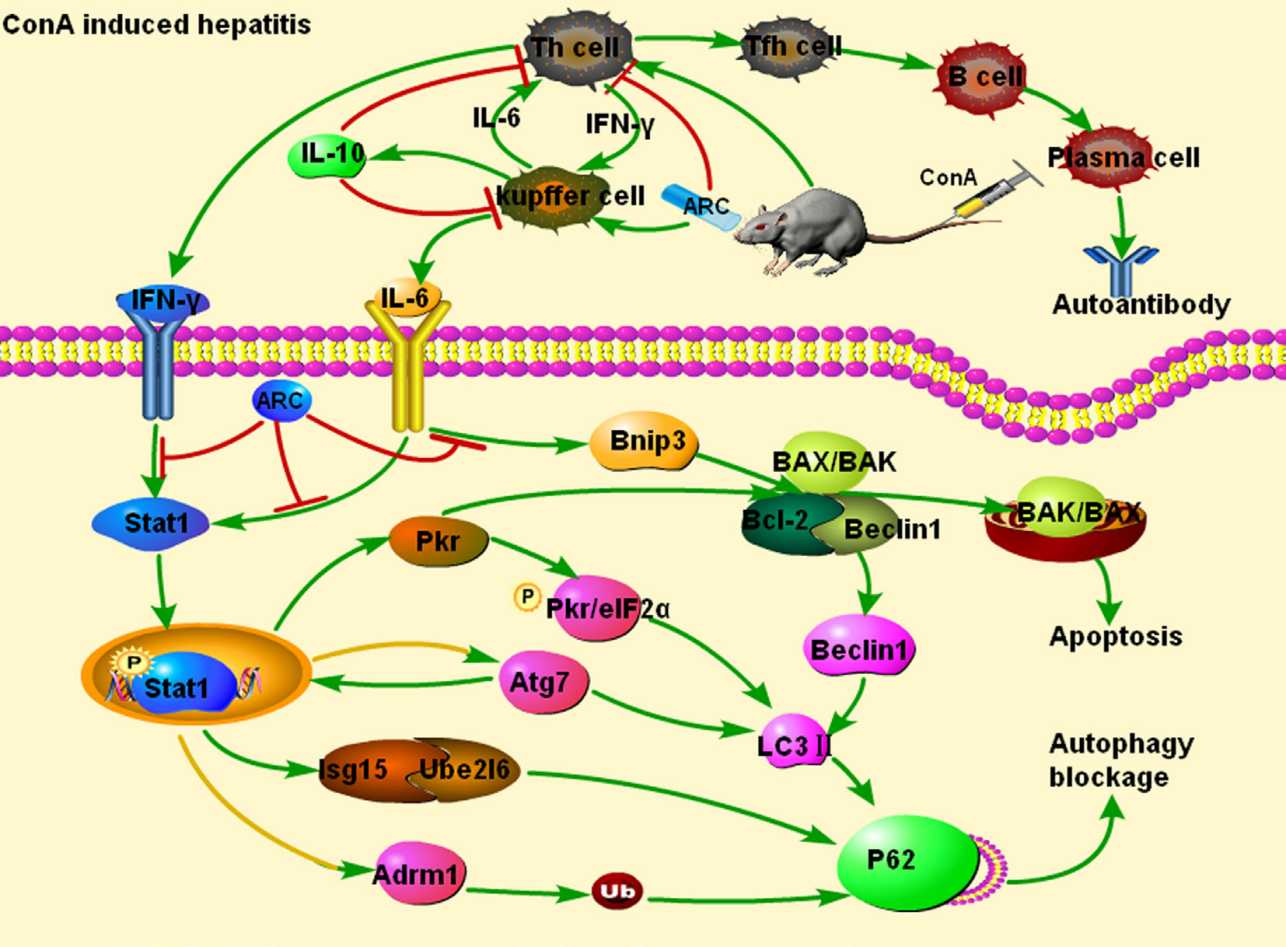
Protective mechanism of ARC on ConA-induced hepatitis. ARC exhibited protective effects through decreasing the levels of pro-inflammatory IFN-γ and IL-6, and increasing the ones of IL-10. The upregulations of Atg7, Beclin 1, and Pkr induced the increase of the expression of LC3 II. The Adrm1, Isg15, Ube2l6, and LC3 II led to the increased expression of p62. ARC pretreatment inhibited autophagy as well as apoptosis by suppressing IFN-γ/IL-6/Stat1 signaling and IL6/Bnip3 signaling. But, whether the upregulations of Atg7 and Adrm1 depend on Stat1 need further study. The green arrows represent activation, the red lines represent inhibition, and the yellow lines with green arrows represent the predictive activation.

All these data suggested that ARC exhibited protective effects through downregulating the levels of IFN-γ and IL-6, upregulating the ones of IL-10. The detailed mechanism of action was shown in Figure 10. The downregulations of Atg7, Beclin1, and LC3 II indicated that the ARC pretreatment inhibited the accumulation of autophagosome at the initial step, while the downregulation of LC3 II and p62 demonstrated that ARC pretreatment alleviated the blockage of autophagy flux. Pkr and Bnip3 mediated both apoptosis and autophagy, ARC pretreatment inhibited both of them. The expression and activation of Stat1 were decreased in ARC pretreatment group. ARC inhibited autophagy as well as apoptosis by suppressing IFN-γ/IL-6/Stat1 signaling and IL6/Bnip3 signaling. The previous study of luciferase activity test showed that ARC could inhibit the IFN-γ/Stat1 and IL-6/Stat3 signaling (122), while present study demonstrated the inhibitions of both IFN-γ/IL-6/Stat1 signaling and IL6/Bnip3 signaling contributed to the protective effects of ARC against ConA-induced hepatitis.

As reported, IFN-γ could induce activated but insufficient autophagy that contributed to p62-dependent apoptosis in epithelial cells (123). A blockage of autophagic flux at level of lysosome results in autophagy-dependent cell death in glioma cells (124). ConA/IFN-γ could trigger autophagy-related necrotic hepatocytes death through IFN-γ-related Irgm1-mediated lysosomal disruption (125). It was suggested that the lysosome might be disrupted in ConA-induced hepatitis. The decreased expression of LC3II and p62 demonstrated that ARC pretreatment alleviated the blockage of autophagy flux by inhibition of the accumulation of autophagosome. However, the effects of ARC on degradation ability of lysosome need further study.

Recent evidences suggested that regulation of autophagy might be a potential strategy for viral hepatitis. HBV and HCV could induce accumulations of autophagosome and p62 but impair lysosomal acidification, leading to incomplete autophagy in autophagic degradation (126, 127). HCV-induced oxidative stress triggered p62-dependent autophagy and interplayed with exosomes to mediate the release of HCV particles (128, 129). Epigallocatechin-3-gallate inhibited HBV replication by resisting insufficient autophagy and enhancing lysosomal acidification (130). The inhibition of autophagosome formation by 3-MA or siRNA targeting Beclin1 and Atg5 markedly inhibited HBV replication (131). Downregulation of the autophagy-related gene expressions by mycophenolic acid could also inhibit HCV replication (132). The autophagy inducer, rapamycin enhanced HBV replication (133), while an autophagy inhibitor chloroquine reduced viral as well as ALT levels (134). In addition to the activation of immune system, the dysregulated autophagy could be a suitable proof for ConA-induced hepatitis model to mimic virus hepatitis. It might be a perfect model for forecasting the antivirus effect of agents. The agents that exerted protective effects on ConA-induced hepatitis through regulating autophagy might also have potentials to be antivirus drugs. Some previously reported agents seemed to support this hypothesis, such as quercetin (24, 135), Shikonin (28, 136), and epigallocatechin-3-gallate (29, 130).

The clinical treatments of the AIH and virus hepatitis are contradictory in clinical. The AIH need immunosuppressive therapy, but it will weaken the ability of antiviral immune suppression, increase the replication HBV or HCV (137). Among the treatments of viral hepatitis, IFN is the preferred one. It eliminates the virus by enhancing immune function. This process will induce or aggravate AIH, especially in HCV infection (138). Regulation of autophagy can simultaneously protect cells from apoptosis when killing the virus. It may be a new strategy for the treatment of immune hepatitis especially when the AIH and virus hepatitis occur simultaneously. Here, the protective mechanism of ARC on ConA-induced hepatitis indicated that it might be a candidate drug for both viral hepatitis as well as AIH. Furthermore, the antiviral effect of ARC has been already confirmed (44–46), but the effects on viral hepatitis need further validation.

With the help of proteomic analysis, it was demonstrated that both autophagy and apoptosis have important clinical implications for the treatment of liver disease. ARC exhibited protective effects on ConA-induced hepatitis by regulating both autophagy and apoptosis. This study indicated the therapeutic potential of ARC as a new strategy for the treatment of immune-mediated hepatitis.

## Ethics Statement

This study was carried out according to the National Institutes of Health Guidelines for the Care and Use of Laboratory Animals and was approved by the Animal Care and Use Committee of Nanjing University, China.

## Author Contributions

JY, GZ and QF designed the study. QF, WX, GZ, JL, XL and TZ conducted the experiments. QF performed analysis and construction of network pharmacology. NZ performed proteomic analysis. QF and JY analyzed the data. QF, NZ and JY wrote the paper.

## Conflict of Interest Statement

The authors declare that the research was conducted in the absence of any commercial or financial relationships that could be construed as a potential conflict of interest.
