# Supplementary figures and images for: Quantitative Proteomic Analysis Reveals That Arctigenin Alleviates Concanavalin A-Induced Hepatitis Through Suppressing Immune System and Regulating Autophagy
Source: Front Immunol. 2018 Aug 16;9:1881. doi: 10.3389/fimmu.2018.01881 (PMC6109684; doi:10.3389/fimmu.2018.01881)

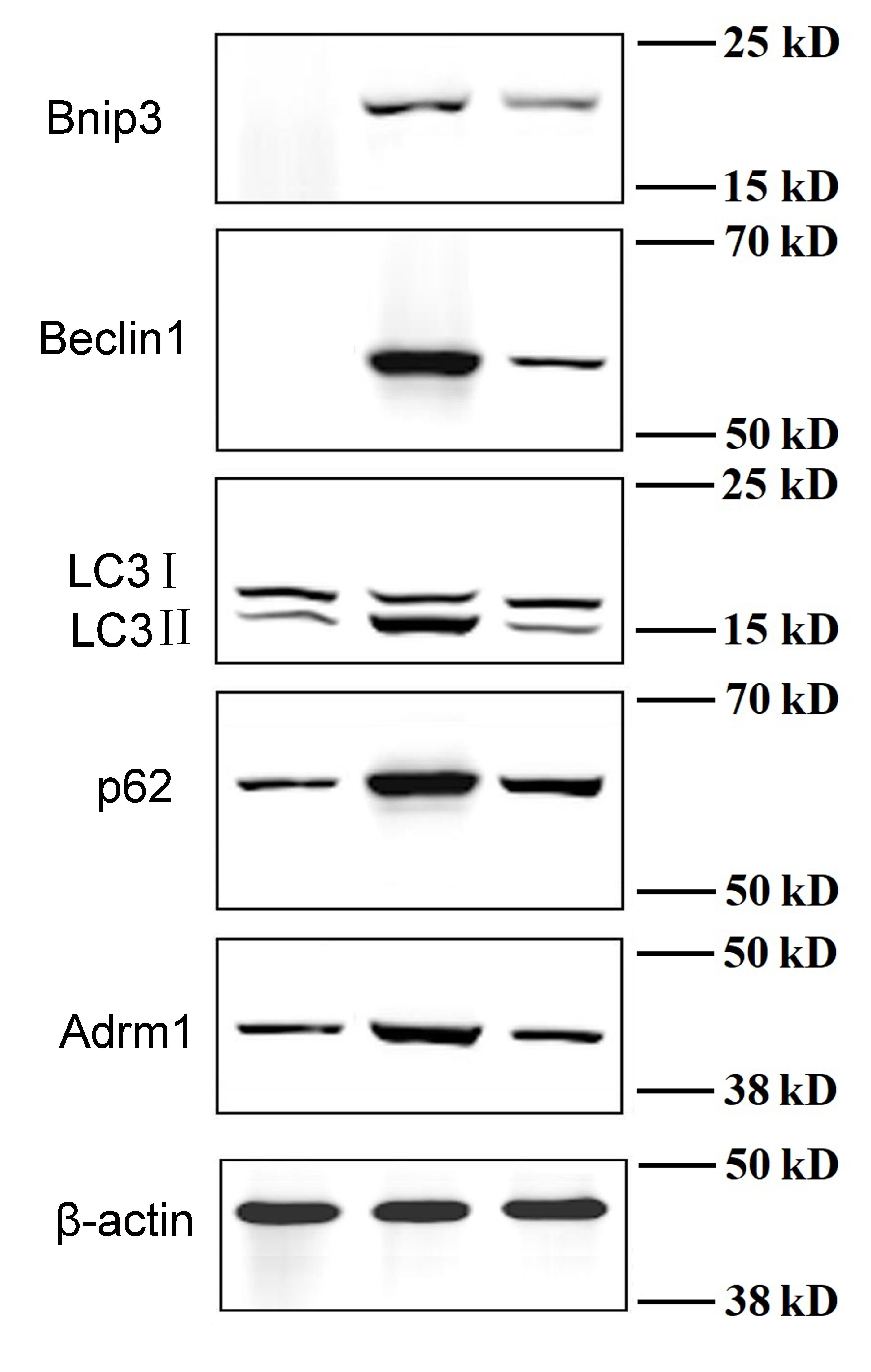

Supplement: Supplementary file 1 [file Image_1.JPEG]

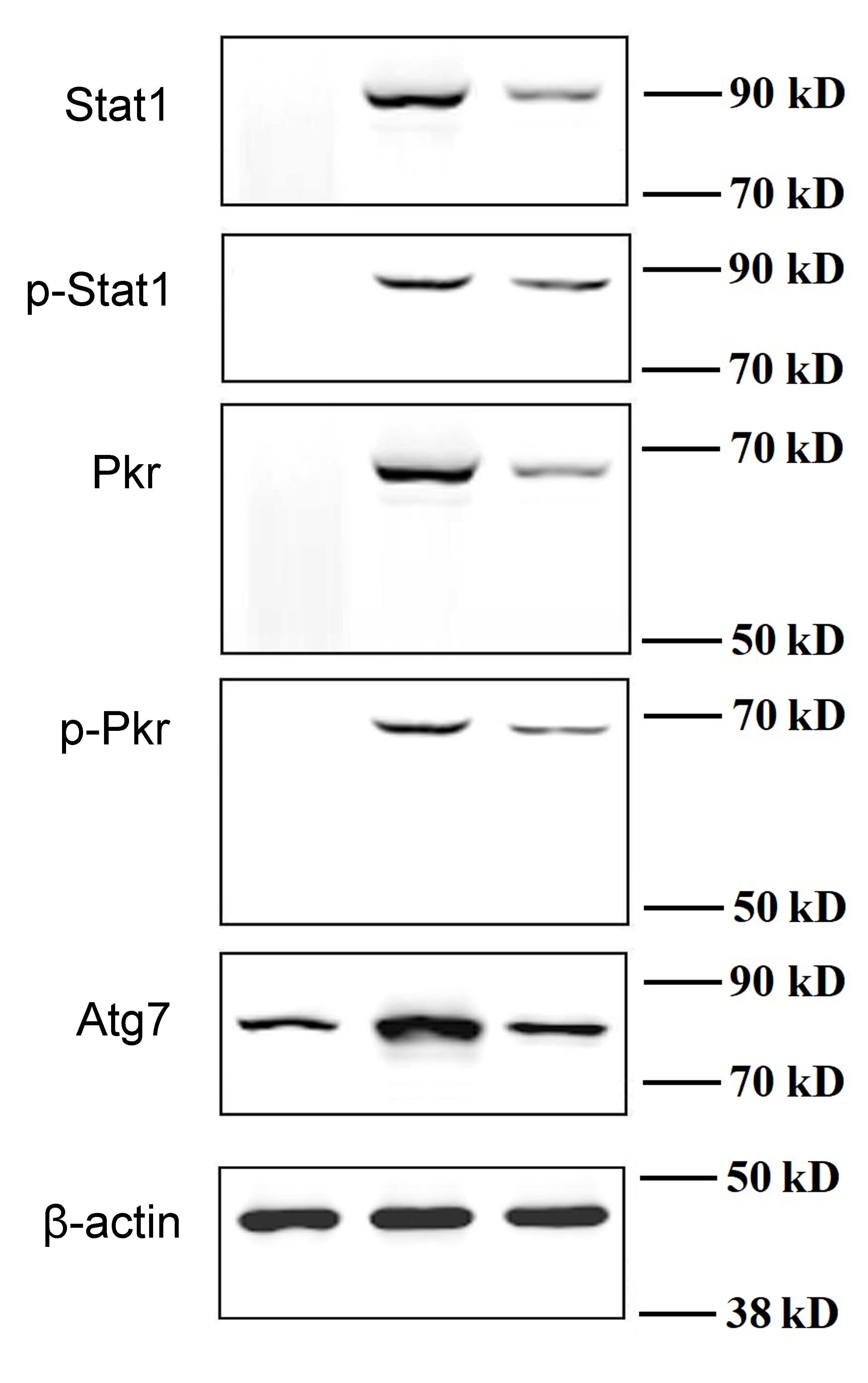

Supplement: Supplementary file 2 [file Image_2.JPEG]
